# Plasmon-Enhanced Photoelectrochemical Current and Hydrogen Production of (MoS_2_-TiO_2_)/Au Hybrids

**DOI:** 10.1038/s41598-017-07601-1

**Published:** 2017-08-03

**Authors:** Ying-Ying Li, Jia-Hong Wang, Zhi-Jun Luo, Kai Chen, Zi-Qiang Cheng, Liang Ma, Si-Jing Ding, Li Zhou, Qu-Quan Wang

**Affiliations:** 1 Key Laboratory of Artificial Micro- and Nano-structures of the Ministry of Education, and School of Physics and Technology, Wuhan University, Wuhan, 430072 P. R. China; 2 Institute of Biomedicine and Biotechnology, Shenzhen Institutes of Advanced Technology, Chinese Academy of Sciences, Shenzhen, 518055 P. R. China; 3 The Institute for Advanced Studies, Wuhan University, Wuhan, 430072 P. R. China

## Abstract

Three component hybrid (MoS_2_-TiO_2_)/Au substrate is fabricated by loading plasmonic Au nanorods on the MoS_2_ nanosheets coated TiO_2_ nanorod arrays. It is used for photoelectrochemical (PEC) cell and photocatalyst for hydrogen generation. Owing to the charge transfer between the MoS_2_-TiO_2_ hetero-structure, the PEC current density and hydrogen generation of TiO_2_ nanoarrays are enhanced 2.8 and 2.6 times. The broadband photochemical properties are further enhanced after Au nanorods loading. The plasmon resonance of Au nanorods provides more effective light-harvesting, induces hot-electron injection, and accelerates photo-excited charges separation. The results have suggested a route to construct nanohybrid by combining one-dimensional arrays and two-dimensional nanosheets, meanwhile have successfully utilized plasmonic nanorods as a sensitizer to improve the photochemical properties of the semiconductor nanocomposite.

## Introduction

As a member of layered two-dimensional material, molybdenum disulfide (MoS_2_) is promising for the applications in energy and environment^1–10^. The MoS_2_ nanosheets could be achieved by break the interlayer van der Waals forces. The band gap of MoS_2_ nanosheets is seriously depended on its layer number, which is varied from 1.3 (bulk) to 1.8 eV (monolayer)^11–14^. Therefore, the few-layered MoS_2_ could be used as an efficient visible light harvester. Meanwhile, the two-dimensional structure provides large contact interface and efficient charge transfer, as a result, the layered MoS_2_ nanosheets have been regarded as a low-cost co-catalyst candidate resently^15–22^. TiO_2_ is a wide band gap (3.6 eV) semiconductor and has exhibited potential in photoelectrochemical (PEC) water splitting and photocatalytic applications^23–29^. The narrow band gap of MoS_2_ can broaden the visible-light response. Additionally, the interface charge transfer between MoS_2_-TiO_2_ hetero-junction would accelerate the charge separation and enhance photocatalytic activity and increase the hydrogen generation^28–36^.

Gold nanoparticles (NPs) supporting tunable surface plasmon resonance in a wide region have been used for various light-matter interaction enhancement^37–41^, in which the main mechanism are broadening light-harvesting region and facilitating the charge separation^42–47^. Yung-Jung Hsu *et al*. have reported that the hot electrons in Au NPs can get over the Schottky barrier and be injected into the conduction band of the TiO_2_, which would supply additional charge carriers for photocatalytic reaction^48^. Xing-Hua Xia *et al*. also reported an efficient water splitting hydrogen evolution reaction of Au nanorods/MoS_2_ nanosheets hybrids through increase the carrier density in MoS_2_ by Au nanorods^49^.

In this paper, we report a three component hybrid (MoS_2_-TiO_2_)/Au including two-dimensional MoS_2_ nanosheets, self-ordered TiO_2_ nanorod arrays, and plasmonic Au nanorods. The microscopic structures and optical properties of (MoS_2_-TiO_2_)/Au are characterized. The photochemical activities of TiO_2_, MoS_2_-TiO_2_, and (MoS_2_-TiO_2_)/Au are comparatively investigated. The physical mechanisms of enhanced light-harvesting, hot electrons injection, and acceleration of separation of photo-excited charges are further discussed.

## Results and Discussion

**Figure 1:**
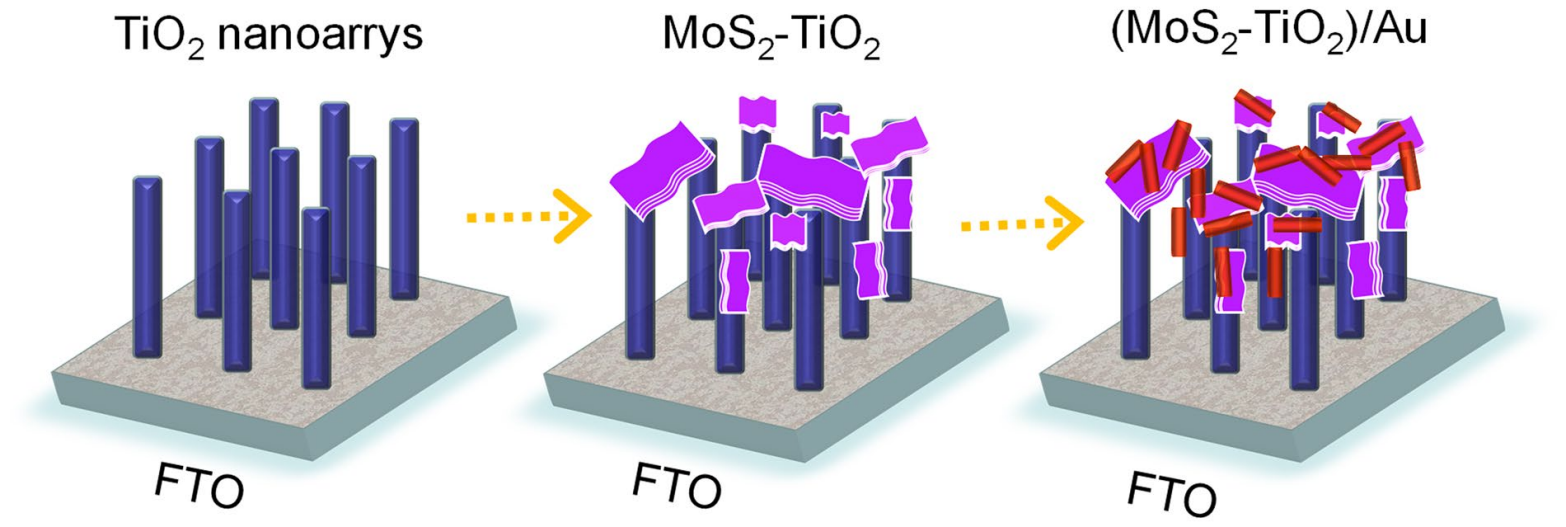
Schematic illustration of preparing (MoS_2_-TiO_2_)/Au nanocomposites.

**Figure 2:**
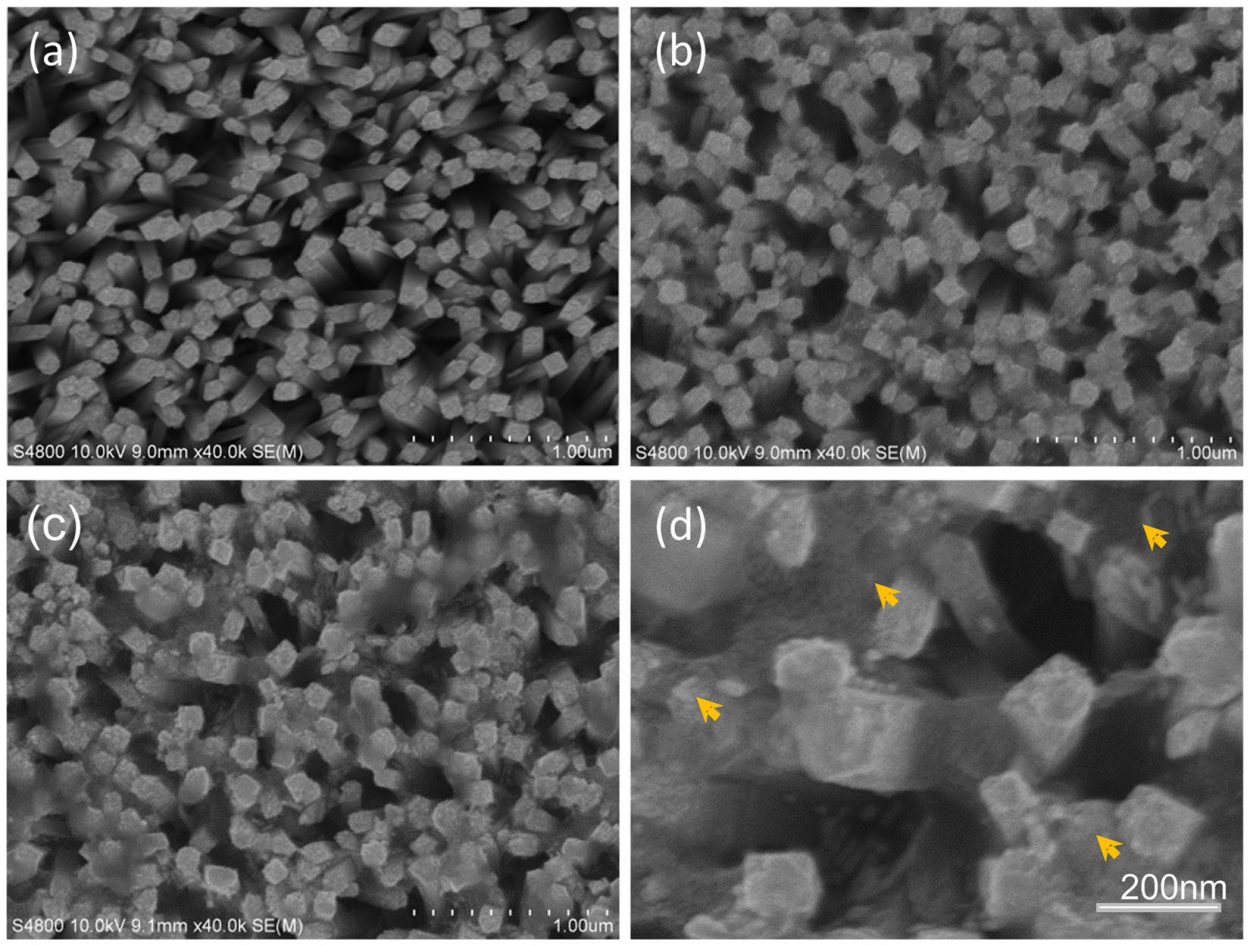
Microscopic structure evolution in prepration of (MoS_2_-TiO_2_)/Au. (**a**–**d**) Top-view SEM images of TiO_2_ (**a**), MoS_2_-TiO_2_ (**b**), and (MoS_2_-TiO_2_)/Au (**c**,**d**). The arrows in (**d**) indictae the locations of Au nanorods.

The preparation procedure of (MoS_2_-TiO_2_)/Au is shown in Fig. 1. The TiO_2_ nanorod arrays are firstly grown on the conductive FTO glass substrate. Then layered MoS_2_ nanosheets are deposited onto the TiO_2_. Finally, the as-prepared Au nanorods are introduced by a drop-casting method. As shown in Fig. 2a, the TiO_2_ nanorods are vertically grown from the FTO conductive glass. The average lateral dimension of TiO_2_ NRs is about 80 nm. Figure 2b shows the sheet-shaped MoS_2_ cover up the top of TiO_2_ nanorods and are also grown into the interspace of nanorod array. The estimated side-length of MoS_2_ nanosheets is in the range from hundreds of nanometers to micrometer-scale. The dimension and amount of MoS_2_ nanosheets can be controlled by the deposition reaction time. As the magnification TEM image shown in Fig. 2d, the locations of Au nanorods are randomly distributed on the MoS_2_-TiO_2_, including on the basal plane of MoS_2_ nanosheets, on the top-end and side-surface of TiO_2_ nanorods, and even on the junction of MoS_2_-TiO_2_.

**Figure 3:**
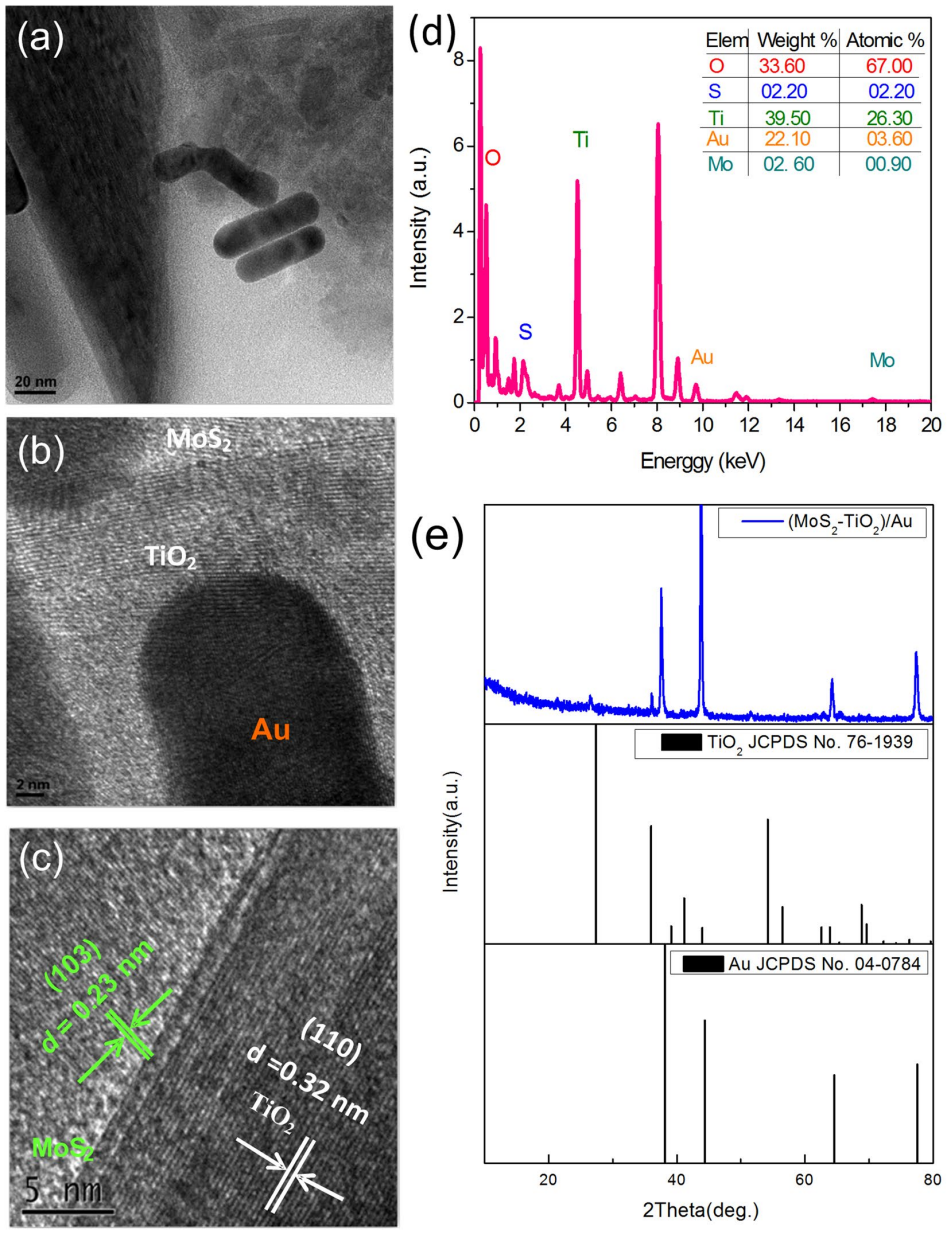
Component analysis of the (MoS_2_-TiO_2_)/Au composites. (**a**–**c**) HRTEM images (**d**) EDX and (**e**) XRD pattern of (MoS_2_-TiO_2_)/Au composites.

For verifying the component in the hybrids, the HRTEM images and EDX analysis of the (MoS_2_-TiO_2_)/Au composites are shown in Fig. 3. The samples are extracted from the FTO glass and placed on the copper grids for TEM observation. The observed Au nanorods have the transverse size of 15 nm and the aspect ratio in the range of 3–4. The lattice fringes of an individual TiO_2_ nanorod with a spacing of 0.32 nm can be assigned to the (110) lattice planes of rutile TiO_2_. The MoS_2_ nanosheets show the lattice fringes with 0.23 nm spacing, corresponding to the (103) planes of MoS_2_. The EDX analysis of the prepared (MoS_2_-TiO_2_)/Au is presented in Fig. 3d. The composite mainly contains Ti and O, and the rest of the trace elements are S, Au, and Mo. The atomic ratios of Mo: S and Ti: O are both approximately 1: 2. In the XRD pattern (Fig. 3e), two sets of diffraction peaks are present, which are assigned to the TiO_2_ nanorod array phase (JCPDS No. 76-1939) and Au nanorods phase (JCPDS No. 04-0784).

**Figure 4:**
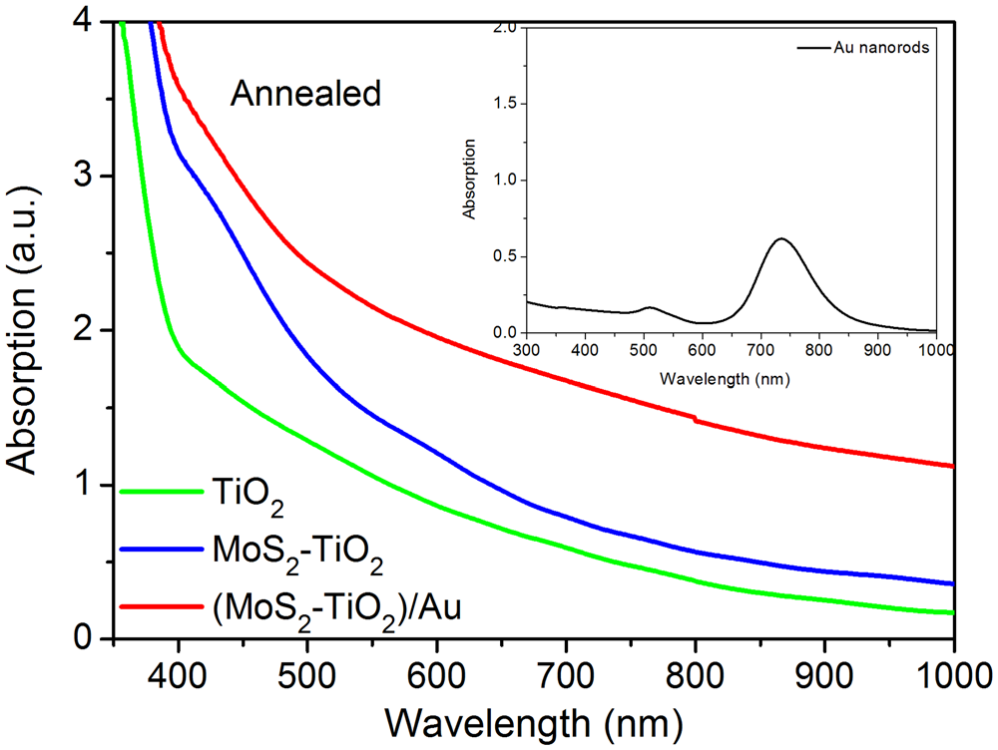
Absorption spectra of pure TiO_2_, MoS_2_-TiO_2_, and (MoS_2_-TiO_2_)/Au. The inset shows the absorption spectrum of as-prepared AuNRs.

Figure 4 displays the absorption spectra of pure TiO_2_, MoS_2_-TiO_2_, and (MoS_2_-TiO_2_)/Au. Pure TiO_2_ only absorbs UV light and exhibits an intense absorption edge before 400 nm, attributing to its band gap of 3.2 eV. The few-layered MoS_2_ nanosheets are reported to have two absorption bands near 400 nm and 600 nm in the visible region^36^, which are shown in the spectrum of MoS_2_-TiO_2_. In the experiment, the sample was tuned to yellowish-brown color when the MoS_2_ nanosheets were grown onto the TiO_2_. These results indicate the deposited MoS_2_ nanosheets have efficient light-harvesting in visible region. The absorption intensity around 700 nm of (MoS_2_-TiO_2_)/Au is obviously enhanced, which is attributed to the plasmon of Au nanorods.

**Figure 5:**
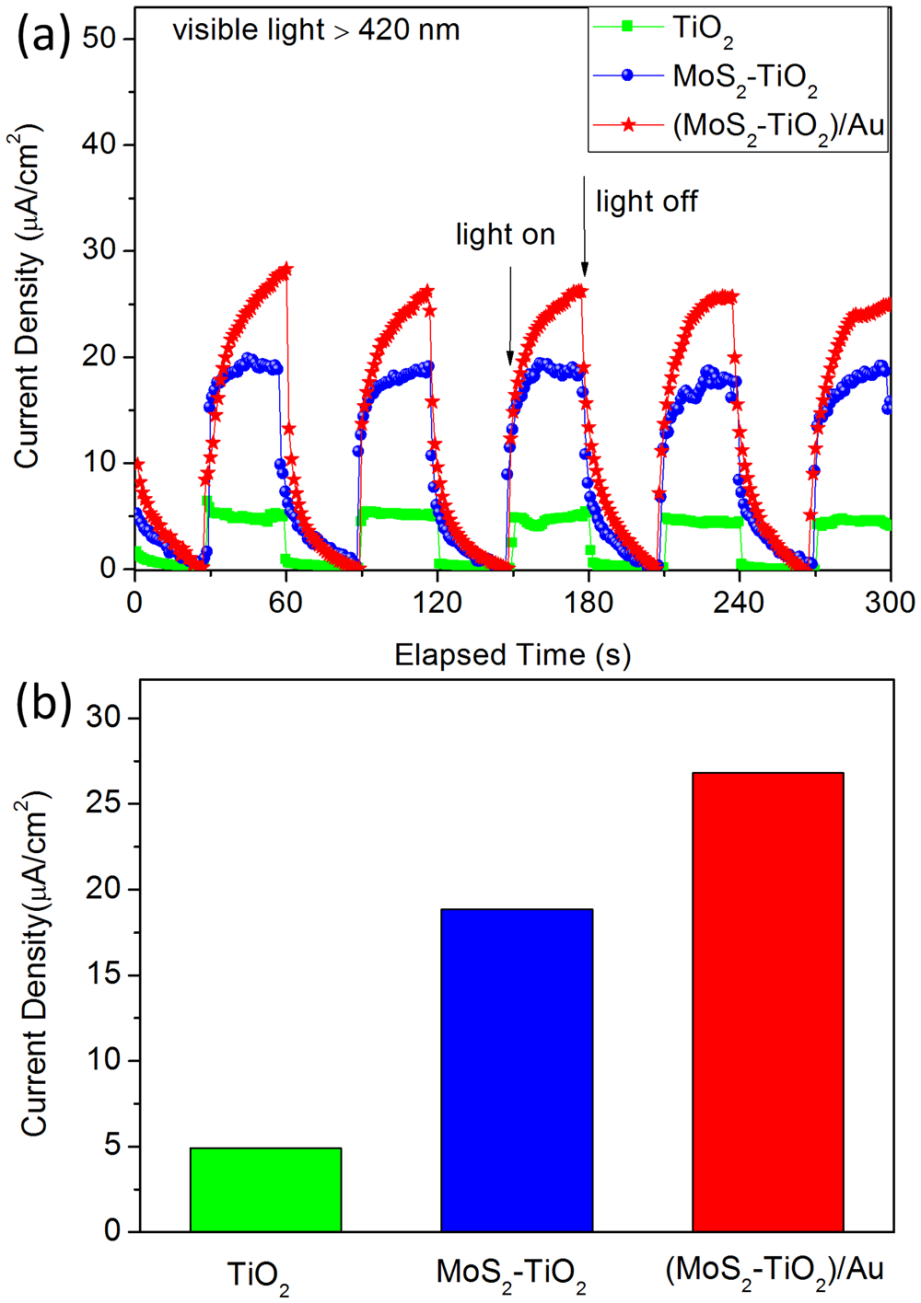
Photocurrent responses of TiO_2_, MoS_2_-TiO_2_, (MoS_2_-TiO_2_)/Au electrodes recorded in 0.1 M Na_2_SO_3_ and Na_2_S aqueous solution under visible light by light-on and light-off cycles.

The photon-electron conversion performance was performed by measuring the photocurrent response of three-electrode PEC cells with the hybrids as photoanode. Figure 5a shows the PEC *I*−*t* curves of the TiO_2_, MoS_2_-TiO_2_, and (MoS_2_-TiO_2_)/Au under the visible-light irradiation (wavelength >420 nm) with a bias of 0.6 V versus Ag/AgCl reference electrode. The electrolyte including Na_2_SO_3_ and Na_2_S solution can consume photo-excited holes on the photoanode. The photo-excited electrons are migrated to the Pt counter electrode through external bias circuit. As the arrows indicated in Fig. 5a, the light irradiation is switched ON/OFF for assessing the photocurrent responses. The average photocurrent densities of the three samples are plotted as bar charts in Fig. 5b. The current densities are 4.9, 18.9, 26.8 μA/cm^2^, for the samples of TiO_2_, MoS_2_-TiO_2_, (MoS_2_-TiO_2_)/Au, respectively. The current density of (MoS_2_-TiO_2_)/Au is 5.5 times that of TiO_2_ and 1.42 times that of MoS_2_-TiO_2_.

The pure TiO_2_ electrode shows a considerably low photocurrent density, because TiO_2_ has large band bap and only responds to UV light. The enhanced photocurrent response of MoS_2_-TiO_2_ electrode can be understood through two aspects of enhanced visible light absorption and accelerated photo-excited charge separation. As discussed in Fig. 4, MoS_2_ nanosheets exhibit efficient light absorption in visible region. The jungle-typed microstructure of TiO_2_ nanorod arrays could trap the incident light inside the arrays through multiple scatterings/reflections and guide the light pass through the MoS_2_ nanosheets multiply times, enhancing the visible light-harvesting. In addition, the band alignment between MoS_2_ and TiO_2_ is favorable for the electron transfer from the conduction band (CB) of MoS_2_ to the CB of TiO_2_ and suppresses the photogenerated carrier recombination of TiO_2_ effectively. Moreover, the inserted MoS_2_ nanosheets connect neighboring TiO_2_ nanorods and act as bridge routes which benefit the electron transfer along the TiO_2_ channel to the conductive substrate.

The highest photocurrent of (MoS_2_-TiO_2_)/Au electrode is benefits from the plasmon-enhanced light absorption and the plasmon-induced hot electron injections. In detail, the Au nanorods work as a reaction sensitizer and enhance the visible light absorption ability of MoS_2_. On the other hand, the Au nanorods have intense plasmon absorption and the plasmon-produced energetic electrons in the (MoS_2_-TiO_2_)/Au nanosystem could also contribute to the photon-to-electron conversion. The hot electrons can get over the Schottky barrier and be injected into the CB of MoS_2_ and TiO_2_.

**Figure 6:**
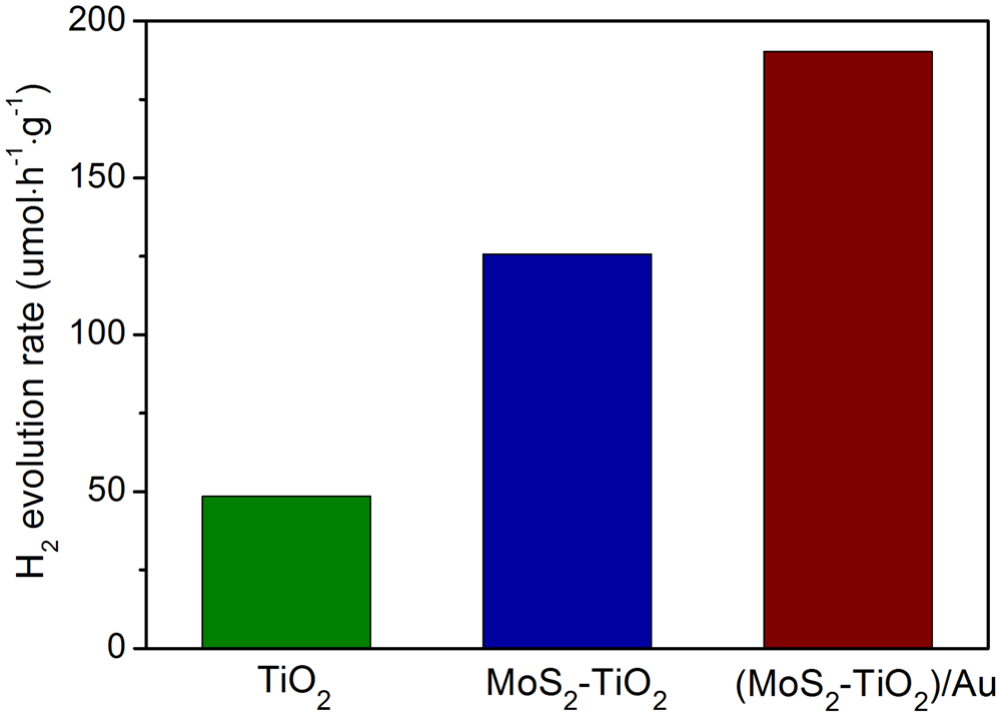
Photocatalytic hydrogen production activities of TiO_2_, MoS_2_-TiO_2_, (MoS_2_-TiO_2_)/Au electrodes under full spectrum in the aqueous solution containing 20% methanol as sacrificial agents.

**Figure 7:**
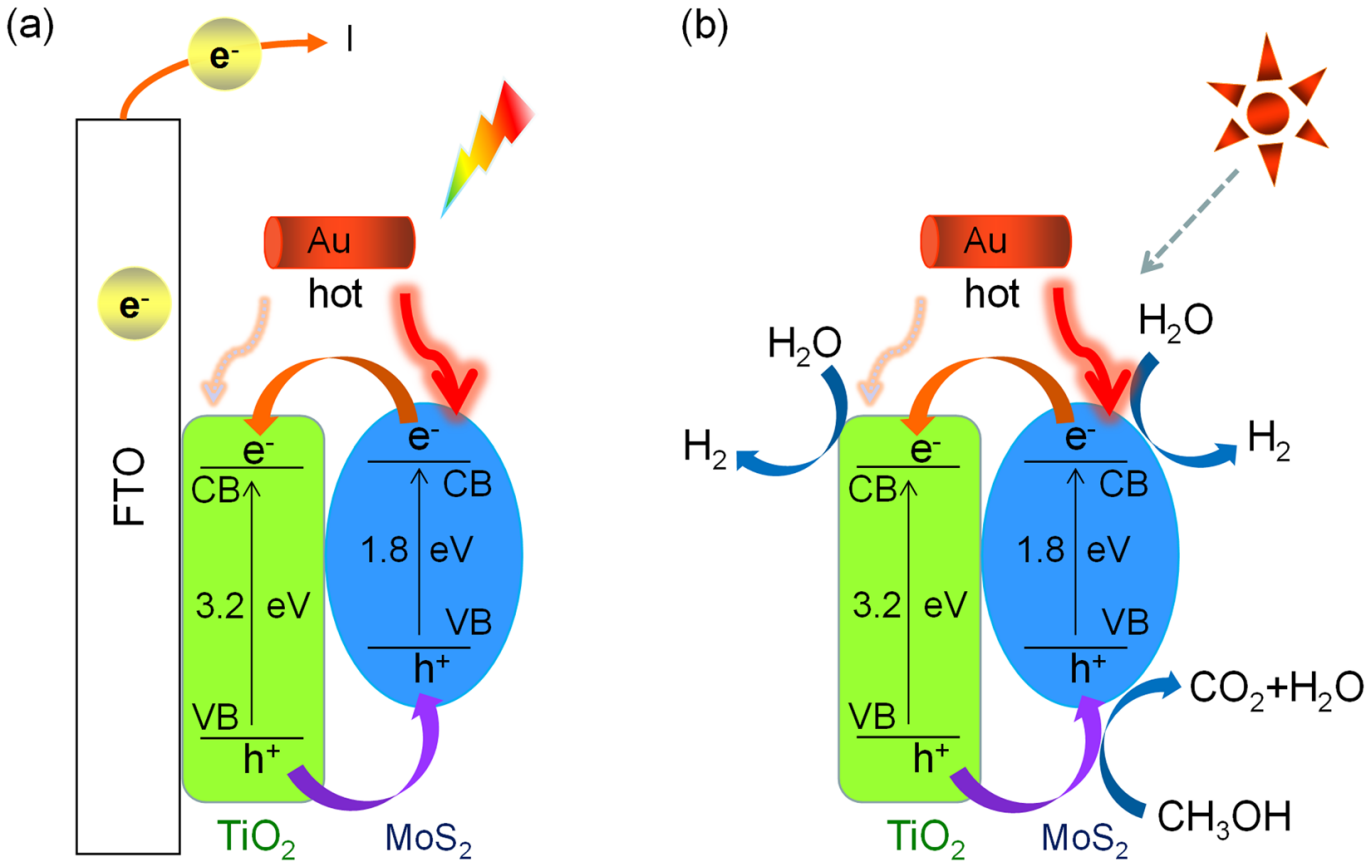
Schematic illustration of energy band structure and electron-hole separation of (MoS_2_-TiO_2_)/Au nanocomposites in PEC cell (**a**) and in photocatalytic hydrogen generation (**b**).

Finally, the hot electron injection of Au nanorods, the enhanced visible light-harvesting and the accelerated charge separation in the (MoS_2_-TiO_2_)/Au hybrids is further demonstrated by testing the photocatalytic hydrogen generation. The H_2_ evolution rate of TiO_2_ and MoS_2_-TiO_2_ under visible light are barely observed, while that of (MoS_2_-TiO_2_)/Au is enhanced. Figure 6 shows the photocatalytic hydrogen generation under full spectrum, TiO_2_ alone shows a low photocatalytic activity with the H_2_ evolution rate of 48 μmol·h^−1^·g^−1^ because of the rapid recombination of electron-hole pairs. The introduction of MoS_2_ results in a significant improvement of photocatalytic H_2_ evolution rate to 125 μmol·h^−1^·g^−1^. The MoS_2_-TiO_2_ composite photocatalysts show enhanced photocatalytic activity because the layered MoS_2_ can help the charge separation also act as an efficient co-catalyst for H_2_ generation than TiO_2_. In the presence of a small amount of Au nanorods in the hybrid photocatalysts, the photocatalytic H_2_ evolution rate of (MoS_2_-TiO_2_)/Au hybrids is further enhanced to 190 μmol·h^−1^·g^−1^. The experimental result of photocatalytic hydrogen generation is consistent with that of the photocurrents under visible light. Figure S1 shows the photocatalytic hydrogen generation under visible light. The corresponding energy band structure and electrons transfer mechanism is schematically shown in the Fig. 7.

## Conclusion

In conclusion, we have prepared a composite of (MoS_2_-TiO_2_)/Au consisting of two-dimensional MoS_2_ nanosheets, self-ordered TiO_2_ nanorod arrays, and plasmonic Au nanorods. Acting as photoanode of PEC cells and photocatalysts for hydrogen generation, the current density of TiO_2_ is increased 2.8 times and the hydrogen generation rate is increased 2.6 time via the charge transfer from MoS_2_ nanosheets. Moreover, the PEC current density and hydrogen generation rate of MoS_2_-TiO_2_ is further enhanced 42% and 52% by plasmon resonance of Au nanorods. The intimate and large contact interface between AuNRs and MoS_2_-TiO_2_ leads to the efficient injection of hot electron, which plays a key factor in determining the high photocurrent response of (MoS_2_-TiO_2_)/Au. The efficient visible light absorption and the high carrier mobility of layered MoS_2_ nanosheets contribute the photocurrent response. In addition, the array-typed nanostructure can effectively trap incident light and the MoS_2_-TiO_2_ hetero-junction can lead to efficient photo-excited charge separation.

## Methods

### Materials Synthesis

Titanium butoxide (TBT, ≥99%), hydrochloric Acid (HCl, 37%), sodium molybdate (Na_2_MoO_4_∙2H_2_O, ≥99%), thioacetamide (TAA, ≥99%). All chemical materials were used without further purification.

### Synthesis of TiO_2_ Nanorod Arrays on FTO

TiO_2_ nanorod arrays were fabricated on the FTO substrate through a hydrothermal method^50^. Before modification, the FTO substrates were washed with acetone, ethanol in an ultrasonic washer for 5 minutes. Then, 0.4 g of titanium butoxide was dissolved into 30 mL of 6 M HCl aqueous solution and then transferred into a Teflon-lined steel autoclave with a capacity of 50 mL. The FTO substrates were placed against the Teflon wall with the FTO side facing down. The autoclave was heated in an oven at 150 °C for 6 h and then cooled down to room temperature. The TiO_2_ nanorods were cleaned with deionized water and ethanol.

### Synthesis of MoS_2_ on TiO_2_

0.3 g of sodium molybdate (Na_2_MoO_4_ · 2H_2_O) and 0.6 g of **thioacetamide** were added. After stirring for 5 minutes, the reaction solution was transferred into a 50 mL Teflon-lined stainless steel autoclave and kept in an electric oven at 200 °C for 10 h. The autoclave was then cooled down to room temperature in the oven.

### Synthesis of (MoS_2_-TiO_2_)/Au

Au nanorods of various aspect ratios were synthesized using a seed-mediated growth method in aqueous solution^51^. Then, the as-prepared Au nanorods were dropped onto the FTO grown with the MoS_2_-TiO_2_ nanocomposites. The samples were thermally treated at 350 °C in N_2_ atmosphere for 0.5 h, and then dried at 70 °C for 10 h.

### Characterizations

Transmission electron microscopy (TEM) and high-resolution TEM (HRTEM) images were taken on a JEOL 2100 F transmission electron microscope at an accelerating voltage of 200 kV. Energy-dispersive X-ray spectra (EDX) analysis was performed on an energy-dispersive X-ray spectrometer incorporated in the HRTEM. Scanning electron microscope (SEM) measurements were carried out with an FEI Sirion 200 scanning electron microscope operated at an accelerating voltage of 10.0 kV. Extinction spectra were recorded by the ultraviolet-visible-near infrared (UV-Vis-NIR) spectrophotometers (TU-1810 and Varian Cary 5000).

### Photoelectrochemical Activity Measurement

A three-electrode configuration was adopted in a quartz cell on the VersaSTAT 3 electrochemical workstation (AMETEK, Inc., United States). A Pt plate and a commercially available Ag/AgCl electrode are used as the counter and reference electrodes respectively, and the sample modified FTO electrode was used as the work electrode. The 0.1 M Na_2_SO_3_ and Na_2_S aqueous solution was prepared to support electrolyte. The effective surface area of the work electrode was 1 × 2.5 cm^2^. Before measurement, the as-prepared samples of TiO_2_, MoS_2_-TiO_2_, (MoS_2_-TiO_2_)/Au were thermally treated at 350 °C in high-purity nitrogen atmosphere for 0.5 h. A 300 W Xenon lamp equipped with an ultraviolet cut-off filter (*λ* > 420 nm) was used as light source.

### Photocatalytic H_2_ Evolution

Before measurement, the samples were dried at 70 °C for 10 h. The photocatalytic hydrogen evolution tests were conducted in a quartz reactor tube with a rubber septum. 20 mg photocatalyst powders were dispersed in 50 mL of aqueous solution containing 20% of methanol as sacrificial reagents. The system was evacuated by using a pump and the reaction solution was stirred for 30 min to remove any dissolved air. The light source was a 300 W Xenon lamp. The temperature of the suspension was maintained by an external water cooling system. The amount of hydrogen gas was automatically analyzed by an online gas chromatography (Tianmei GC-7806).

## Electronic supplementary material

Supplementary Information
