# Supplementary material for: Plasmon-Enhanced Photoelectrochemical Current and Hydrogen Production of (MoS2-TiO2)/Au Hybrids
Source: Sci Rep. 2017 Aug 3;7:7178. doi: 10.1038/s41598-017-07601-1 (PMC5543159; doi:10.1038/s41598-017-07601-1)
Supplement: Supplementary file 1 — Supplementary Information [file 41598_2017_7601_MOESM1_ESM.doc]

Supplementary Information for

**Plasmon-Enhanced Photoelectrochemical Current and Hydrogen Production of (MoS_2_-TiO_2_)/Au Hybrids**

Ying-Ying Li^1,§^, Jia-Hong Wang^1,2,§^, Zhi-Jun Luo^1,§^, Kai Chen^1,3^, Zi-Qiang Cheng^1^, Liang Ma^1^, Si-Jing Ding^1,*^, Li Zhou^1,*^, Qu-Quan Wang^1,3,*^

* Corresponding authors, E-mail: [qqwang@whu.edu.cn](../../../../C:%5CUsers%5Ckc%5CDesktop%5Cqqwang@whu.edu.cn) (Q.-Q. Wang), [zhouli@whu.edu.cn](mailto:zhouli@whu.edu.cn) (L. Zhou), [sjding@whu.edu.cn](mailto:sjding@whu.edu.cn) (S.-J. Ding).


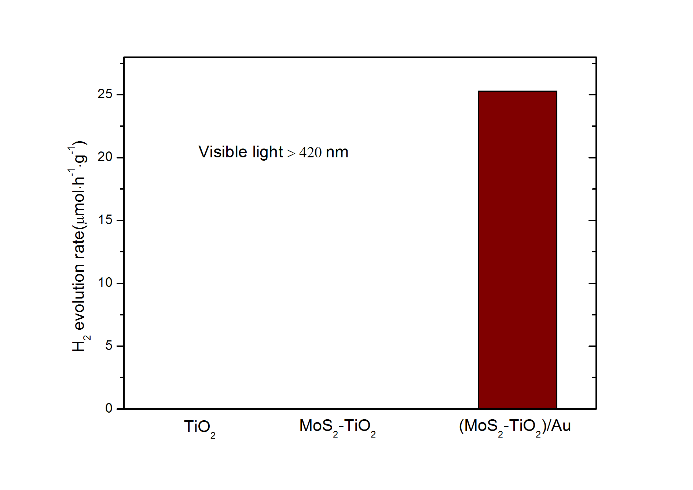


**Figure S1**. Photocatalytic hydrogen production activities of TiO_2_, MoS_2_-TiO_2_, (MoS_2_-TiO_2_)/Au electrodes under visible light in the aqueous solution containing 20% methanol as sacrificial agents.
